# A Unified Local–Global Feature Extraction Network for Human Gait Recognition Using Smartphone Sensors

**DOI:** 10.3390/s22113968

**Published:** 2022-05-24

**Authors:** Sonia Das, Sukadev Meher, Upendra Kumar Sahoo

**Affiliations:** National Institute of Technology Rourkela, Rourkela 769008, India; smeher@nitrkl.ac.in (S.M.); sahooupen@nitrkl.ac.in (U.K.S.)

**Keywords:** gait recognition, multi-scale CNN, smartphone sensor, inertial sensor

## Abstract

Smartphone-based gait recognition has been considered a unique and promising technique for biometric-based identification. It is integrated with multiple sensors to collect inertial data while a person walks. However, captured data may be affected by several covariate factors due to variations of gait sequences such as holding loads, wearing types, shoe types, etc. Recent gait recognition approaches either work on global or local features, causing failure to handle these covariate-based features. To address these issues, a novel weighted multi-scale CNN (WMsCNN) architecture is designed to extract local to global features for boosting recognition accuracy. Specifically, a weight update sub-network (Ws) is proposed to increase or reduce the weights of features concerning their contribution to the final classification task. Thus, the sensitivity of these features toward the covariate factors decreases using the weight updated technique. Later, these features are fed to a fusion module used to produce global features for the overall classification. Extensive experiments have been conducted on four different benchmark datasets, and the demonstrated results of the proposed model are superior to other state-of-the-art deep learning approaches.

## 1. Introduction

Human gait is a biometric attribute that is useful and attracting attention in different fields such as surveillance, biomedical engineering, clinical analysis, etc. Commonly, gait analysis is essential in a clinical investigations such as fall detection [1], rehabilitation [2,3], physical therapy [4], etc., for the well-being of a patient suffering from underlying diseases such as strokes, Parkinson’s, or progressive supranuclear palsy (PSP). Current studies focus on the recent development of human gait rehabilitation therapy based on the state of the brain by employing the brain–computer interface (BCI) system [5,6,7]. BCI systems are capable of decoding the cognitive state of a patient to provide feedback to an external device such as a wheelchair, robotic prostheses/orthoses, or muscle simulator by acquiring brain signals from electroencephalographic (EEG), as discussed in these papers [6,8,9]. In [10], the authors utilized EEG-based brain signals for distinguishing between a healthy person and a patient by measuring the level of attention of a person toward his gait. Furthermore, to measure the attention level, numerous methods have been developed such as the continuous performance test (CPT) and the test of variable attention (T.O.V.A.) referred in [11]. Apart from that, the eye-movement tracking technique [4] is adopted among the PSP patient to improve temporal aspects of the gait of the patient by estimating the eye-movement parameters through a GP3 eye-tracker [12,13].

Although human gait is very familiar in the era of clinical analysis, the current paper exploits this attribute in individual recognition. Generally, gait recognition models are commonly implemented either through vision-based methods, which utilize the video and image data [14,15,16,17,18,19] or through inertial-based devices such as wearable sensors/floor sensors/smartphones’ sensors to capture signals of human movement [20,21,22,23] to infer gait identity. Although the vision-based method has been extensively studied and can achieve a high recognition rate, its application is limited due to the high acquisition cost and difficulty in the deployment of cameras in a real-life environment. On the other hand, inertia-sensor-based technology such as smart devices with built-in sensors, wearable sensors, and smartphones are on excess demand due to its low cost, convenience carrying, and good real-time performance [23,24,25]. Today, smartphones are featured with many inertial sensors such as an accelerometer and gyroscope to capture the speed and direction of a moving person [26,27,28]. Therefore, it is beneficial to track the person in survieillance. Currently, many research studies [24,29,30,31] have been completed in this area, which motivates us to utilize smartphone sensor data for gait recognition.

In this article, an ideal approach is proposed to effectively handle covariate-based gait signals by utilizing multi-scale CNN concepts to get deep spatial features using down-sampled signals referred to as local features. However, the key difference between traditional multi-scale CNN and our proposed approach is to predict features at different scales to obtain discriminant features. To accomplish this task, a branch network called a weight update subnetwork (Ws) is coupled to each CNN to highlight the relevance feature vectors and specify more weights by using the fisher discriminant criterion [32]. The down-sampled signal from low scale to high scale indicates elusive variations between gait poses due to the effect of the covariates. Therefore, a fusion module is implemented to generate the effect of dependencies between low-scale samples to high-scale samples. Eventually, all these weighted features are flattened into a 1D array for producing a single feature vector. In the end, a softmax layer followed by a fully connected network (FCN) is employed to process the feature vectors for final classification.

The main contributions of this article are briefly outlined as follows:

- Inspired by the multi-scale approach, the proposed model leverages multi-scale convolutional neural networks [33], a fusion network, and a weight update sub-network, and it combines them in an end-to-end manner to address the covariate issues.
- In particular, it aims to highlight relevant local features in each scale with respect to label-based gait patterns by incorporating weight update sub-networks (Ws). Furthermore, global features are extracted with the help of a fusion network. The significance of discriminative local and global features is to handle intra-class variations and inter-class variations, respectively.
- The proposed framework has been gone through extensive empirical evaluations using four benchmark gait-based inertial datasets: OU-ISIR, whuGAIT, Gait-mob-ACC, and IDNet, and the results are compared with many state-of-the-art gait recognition models such as IdNet [23], CNN [34], LSTM [30], DeepConv [35], CNN+LSTM [24], and the proposed model outperforms others.

The remainder of this paper is organized as follows. Literature related to the proposed method is discussed in Section 2. The framework of the proposed model and its corresponding architecture is described in Section 3. The experimental setup and results are presented in Section 4 and discussed in Section 5. Section 6 provides the conclusion.

## 2. Related Work

### 2.1. Sensor-Based Gait Identification

Recently, sensors-based gait analysis has become a rapidly growing research platform [21,24,30,36,37,38,39,40]. In early research, Nickel et al. [41] captured accelerometer data through smartphones, where cepstral coefficients are extracted from the data to consider as a feature set, and support vector machine (SVM) has been used for training these features. In 2012, Juefei-Xu et al. [42] developed a step-independent gait identification model from a continuous tracking of smartphone-based acceleration and gyroscope data. Furthermore, several studies have been proposed for handling multi-modal sensor data in gait identification using fusion-based techniques [43], a Gaussian mixture model (GMM-UBM) [42], and CNN methodologies [44].

### 2.2. Deep Learning Approaches on Gait Analysis

In the last few years, several deep learning models have been proposed for gait-based identification [24,30,39,45,46]. For example, convolutional neural networks (CNN) are widely used in many existing gait recognition methods [23,24,47]. IDNet [23] incorporates both a CNN-based deep learning approach and machine learning tools such as SVM to process inertial signals captured from smartphones for gait authentication. Here, the CNN network has been adopted as a universal feature extractor and SVM for gait classification. Another related work of deep learning is multi-scale analysis, which has achieved a series of progress in the field of detection, classification, and identification. So far, a multi-scale strategy has been widely used in deep learning for gait-based recognition [48,49,50] where it explores spatial features at multiple scales and learns more details about different gait regions to extract local features. However, it fails to find dependencies among the spatial features as well as overall gait variations. Gait recognition methods based on global representations deal with gait data as a whole and do not pay attention to local gait details; some examples include GaitNet-1 [51] and GaitNet2 [52], but these methods are sensitive to the covariate factors. To address the above issues, for the first time, in this context, a novel model (WMsCNN-Local-Global) has been proposed to extract more comprehensive features, which contains both local and global information of inertia signals acquired from smartphones.

## 3. System Overview

**Figure 1:**
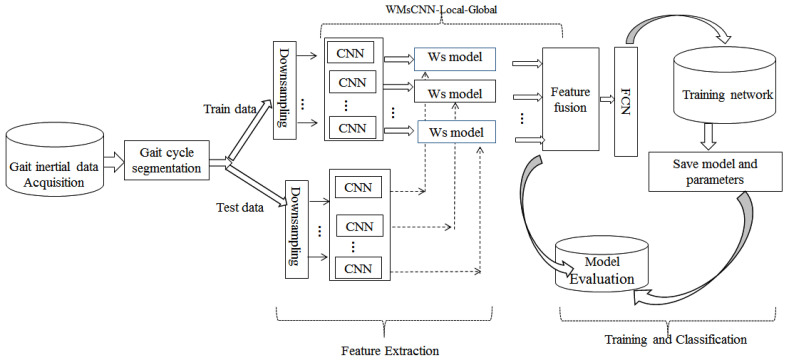
Overview of the proposed framework.

The proposed framework is comprised of five parts: acquisition of inertial gait data, segmentation of gait cycle, deep feature extraction, training, and classification. The schematic diagram of the proposed framework is shown in Figure 1. The acquisition of inertial gait data is done through an accelerometer and gyroscope sensor, which are useful for tracking a person’s movement along the X, Y, and Z directions, denoted as $A_{x},A_{y},A_{z}$, and $G_{x},G_{y},G_{z}$ respectively. All the sensor data are normalized using $L_{2}$ norm to avoid uncertain movements of smartphones such as shifting of smartphones from left to right or up to down positions. Furthermore, the gait cycle segmentation task is carried out using the acceleration data along the X, Y, and Z directions. The paper adopts U-net [24] to perform this task. All the gait cycles are randomly split into a gallery (train) and probe (test) sequences. To obtain deep features from the multi-scale technique, samples are further down-sampled into different time scales and processed through several convolutional layers, which are treated as an independent feature set. A novelty of the proposed method lies in the localization of the important feature map and assigning weights to the feature vector for training and classification. To perform this task, a weight update subnetwork (Ws) is designed to connect each CNN architecture. Later, all the locally weighted features are fused to get dependence among them to utilize for overall gait variations. Eventually, all the fused features are flattened and fed to the fully connected layer for classification.

### 3.1. Proposed Approach

**Figure 2:**
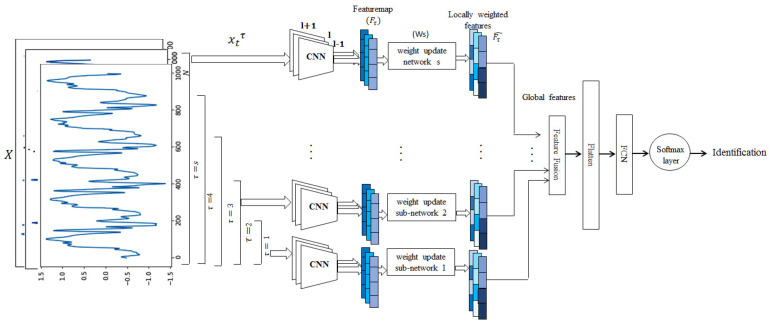
Detailed design of the WMs–CNN–Local–Global model.

The objective of the paper is to estimate the importance of feature vectors with respect to their label prediction and ignore other features that may misguide a classifier. In other words, different weights can be assigned to the local feature vectors from different scales by giving more weight to the representative features and less weight to others. In order to accomplish this, a multi-scale signal is reconstructed from a single scale by down-sampling and further processed through a stack of CNN structures to get deep features at different time scales. The detailed design of the proposed model is shown in Figure 2.

**Multi-scale signal reconstruction:** The inertial data acquired from the accelerometer and gyroscope are simultaneously considered inputs. It can be expressed as $x_{t}=\left[A_{x},A_{y},A_{z},G_{x},G_{y},G_{z}\right]$ at time step *t* along the X, Y, and Z-axis. Combining all the time steps can be represented as a gait cycle $X=[x_{1},x_{2},\ldots ,x_{N}]$, where N is the number of steps to be considered in each gait cycle. Assume each gait cycle ’X’ is down-sampled at a time scale ’τ’ is expressed below.

(1) $$x_{t}^{\tau }=\frac{1}{\tau }\sum_{k=(t-1)\tau +1}^{t\tau }X_{k},1\leq t\leq \frac{N}{\tau },$$

where $x_{t}^{\tau }$ is a down-sampled signal computed by taking an average of consecutive data points *t* of the input signal $X_{k}$ at time index k. The whole expression of the multi-scale signal is denoted as $x^{\tau }=\left\{x_{1}^{\tau },\ldots ,x_{t}^{\tau },\ldots ,x_{N/\tau }^{\tau }\right\}$.

So far, the effectiveness of the convolutional neural network has been proven as a good feature extractor in the field of motion data, image analysis, speech signal processing, etc. [53]. Thus, we are motivated to incorporate CNN architecture into each scaled signal to obtain significant features. Each scaled sub-sample $x_{t}^{\tau }$ is fed to the four convolutional layers of the CNN network, which is followed by a pooling layer. The output of the layer is expressed as below.

(2) $$x_{t}^{l,\tau }=ReLU\left(\sum_{m=1}^{M}W_{m}^{l-1,t}*x_{t+m-1}^{l-1,\tau }+b_{t}^{l}\right),$$

where $x_{t}^{l,\tau }$ denotes the output layer, l∈(1,2,3,4); * denotes the convolutional operator; M is the kernel size; $b_{t}^{l}$ is the bias term at layer *l*; W is the weight of the lth feature map; ReLU denotes an activation function; *m* and *l* denote the index of the kernel and convolutional layer, respectively. Later, by applying a pooling layer, the output local feature is given as

(3) $$f_{t}^{l,\tau }=max\left(x_{t}^{l-1,\tau }\left(n\right)\right),\;\;n\in \left[\left(j-1\right)w,tw\right],$$

where $f_{t}^{l,\tau }$ is the output of the maximum value among the (l−1)th layer obtained from samples $x_{t}^{l-1,\tau }\left(n\right)$, *n* represents the nth output neurons at the jth position of local features, and *w* is the width size of the pooling layer.

**Figure 3:**
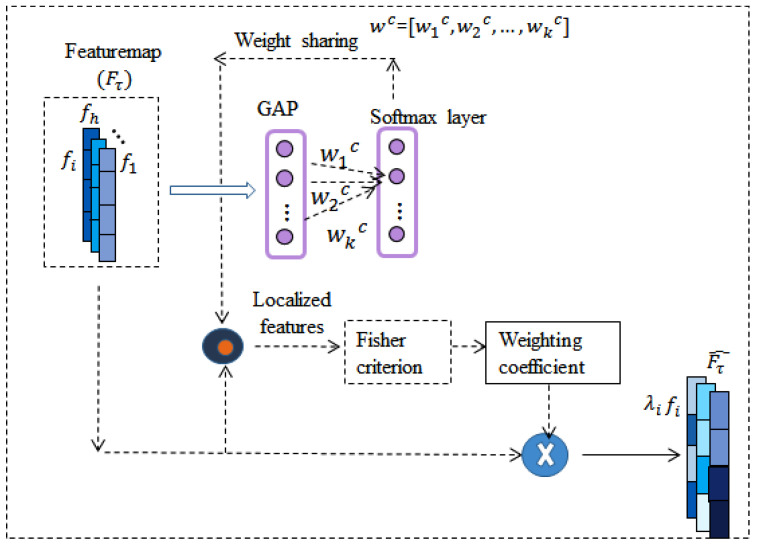
Architecture of a weight update sub-network (Ws) to achieve discriminative features.

**Weight update sub-network (Ws):** The proposed sub-network aims to explore a novel spatial adaptive weighting technique using the Fisher-based discrimination [54] among the feature vectors with respect to their labels. The main idea is to map the classifier weights to each feature vector to perform localized classification. Subsequently, weights are assigned to each feature vector depending on its contribution to its label data. To accomplish this task, a sub-network is inserted between the last CNN layer and a classifier. A global average pooling layer and a soft-max layer are the part of the sub-network that finds localized features for each class label. The architecture of the weight update sub-network (Ws) is shown in Figure 3.

Suppose in multi-scale signal analysis, $F_{\tau ,k}$ represents the output feature map of CNN for each scale of unit *k* after passing through a global average pooling layer (GAP), which is specified as below.

(4) $$F_{\tau ,k}=\frac{1}{h}\sum_{i}f_{i,k},$$

where $f_{i,k}\in R^{c}$, i=1,2,…,h is the local feature vectors at unit *k*. The localized classification is performed using the dot product between the feature vectors and the weights of the classifier, as described in (5).

(5) $$\hat{f_{i}}=\sum_{i}\sum_{k}w_{k}^{c}f_{i},$$

where $\hat{f_{i}}\in R^{N}$ is the localized classification score at class c, $w_{k}^{c}$ is the class-specific weight vector assigned to local features, and ’*i*’ is the location of each feature. Subsequently, weights are updated by projecting the localized classification scores from high-dimensional space to low-dimensional space, based on their intra (within) and inter (between) classes distributions.

Let the localized scores ${\hat{f}}_{i}$ be projected from the *N* dimensional space to $N{}^{\prime }$ dimensional space for separating two different classes. Then, the weight $\lambda_{i}$ is computed by considering an $N{}^{\prime }$ eigenvector corresponding to the maximum eigenvalue given below.

(6) $$\begin{array}{cc} {\hat{\sum }}_{w} & =\sum_{i}\sum_{\hat{f}\in c_{i}}\left(\hat{f}-m_{i}\right)\left(\hat{f}-m_{i}\right){}^{\prime } \end{array}$$

(7) $$\begin{array}{cc} {\hat{\sum }}_{b} & =\left(m_{i}-m\right)\left(m_{i}-m\right){}^{\prime } \end{array}$$

(8) $$\begin{array}{cc} \lambda_{i} & =max_{N{}^{\prime }}\left(eig\left({\hat{\sum }}_{w}^{-1}{\hat{\sum }}_{b}\right)\right), \end{array}$$

where ${\hat{\sum }}_{w}$, ${\hat{\sum }}_{b}$ are the within-class matrix and between-class matrix, which are computed in (6) and (7), respectively. ${\bar{m}}_{i}$ and *m* are the mean of the local and global class, respectively.

### 3.2. Fusion Network

All the locally weighted features from low-scaled gait variations to large-scaled variations are fused to obtain linear dependency among them. As it is a linear combination of discriminative features from small gait sequences to large gait sequences, the resultant feature set is named the global feature set. It is computed as follows.

(9) $${\hat{F}}_{global}=W_{\tau 1}{\hat{F}}_{\tau 1}+W_{\tau 2}{\hat{F}}_{\tau 2}+\ldots +W_{\tau s}{\hat{F}}_{\tau s},$$

where the fusion weights $W_{\tau 1},W_{\tau 2},\ldots ,W_{\tau s}$ are the adaptive parameters learned from the training sets. Subsequently, the global feature ${\hat{F}}_{global}$ is fed to a fully connected layer (FC) and a softmax layer. The output expressions of both the layers are presented below:

(10) $$\begin{array}{c} {\hat{F}}_{global}^{l}=b^{l}+{\hat{F}}_{global}^{l-1}\cdot w^{l} \end{array}$$

(11) $$\begin{array}{c} o=Softmax({\hat{F}}_{global}*W_{0}+b_{0}) \end{array}$$

### 3.3. Training and Classification

The training of the proposed model is performed in an end-to-end manner, learning combined with multiple weight update sub-networks (Ws) and overall networks in a single unified fashion using a backpropagation algorithm. To do so, Ws sub-networks are trained independently from fewer scales to more scales to obtain local optimization under the supervision of label-based gait sample patterns. The total classification loss of the local features is observed below.

(12) $$L_{local}=\sum_{i=1}^{i=s}\alpha_{i}L_{i}\left({\hat{F}}_{i},y\right),$$

where *s* represents the total number of sub-networks in the local module, $\alpha_{i}$ is the weight parameter of the each sub-network, and *y* is the label of gait patterns at different conditions. Then, the overall training is computed at the final layer to obtain global optimization, and the gradients are propagated backwards layer-by-layer to update the weights. The overall loss of the proposed framework (WMsCNN-Local-Global) can be represented by

(13) $$L_{overall}=\alpha L_{Local}\left(F_{i},y\right)+\beta L_{global}\left(F_{global},y\right),$$

where α and β are both weight updated parameters. Each loss function is defined in terms of cross-entropy loss.

The network is iteratively trained through several epochs to update the model using the training set. Furthermore, the training set is split up into distinct batches B, and each batch B has B segments. In each epoch, the training set is shuffled and computes a set of output vectors *O* based on its loss function. Let each vector $o^{i}\in O$ be the estimated prediction score for each label. ${\hat{o}}^{i}\in O$ is the actual score for label *i*. Then, the cross-entropy loss-based classification problem can be formulated as below.

(14) $$L_{B}\left(o,\hat{o}\right)=\frac{1}{B}\sum_{i=1}^{K}{\hat{o}}_{i}lno_{i}+\left(\left(1-{\hat{o}}_{i}\right)ln\left(1-o_{i}\right)\right),$$

where $L_{B}$ the cross-entropy loss function used to update network’s internal parameters through back-propagation [55]. When all the batches have been used to train the network, one training epoch is completed; then, the process is repeated with a new epoch until it meets a stopping condition as referred in Section 4.1. It is observed from (14) that a large difference between $o_{i}$ and ${\hat{o}}_{i}$ results in a high value of entropy loss. Basically, the training network adopted this concept for optimization.

## 4. Experimental Setup and Result Analysis

The experiment is conducted by integrating weight update sub-networks (Ws) into various CNN architectures. All the experiments are implemented using the Keras API and Caffe framework. The proposed network evaluates different challenging datasets having covariate conditions and compares them with several state-of-the-art deep learning approaches, such as CNN, LSTM, CNN+LSTM, IdNet, and Deepconv modules. A brief description of the datasets is given in Table 1.

### 4.1. Different Sensor-Based Gait Dataset

whuGAIT datasets [24]: Here, 118 subjects are taken into consideration in the data collection, out of which 20 subjects have a large number of data, where each holds thousands of samples. The rest of the subjects contain a smaller amount of data, each holding hundreds of samples. Furthermore, each data sample contains a three-axis accelerometer and gyroscope data. Here, all the data are sampled at 50 Hz. The dataset is organized into eight subsets from Dataset #1 to Dataset #8. In this paper, Dataset #1 and Dataset #2 are used for classification, while the rest, Dataset #5–#6 and #7–#8, used for gait authentication and gait data extraction, respectively.

IdNet dataset [23]: It has 50 subjects and collects data from both a tri-axial gyroscope and accelerometer embedded in a smartphone. The sampling rate of the sensor data is 100 Hz. These data include two such variations, such as people wearing different shoe types and different clothes at a different time of gait data acquisition.

OU-ISIR dataset [21]: So far, it is the largest population dataset in terms of capturing inertial-sensor-based gait data. Two types of devices such as 3IMUZ sensors and Motorola ME860 are used to capture the sensor data. The first one captures both accelerometer and gyroscope data, while the second one collects triaxial accelerometer data. Each sensor works at 100 Hz. The experiments are performed on two different sets of users on the basis of two different conditions. One experiment is conducted for evaluation in the presence of a large set of the population around 744 subjects; another one is conducted on 408 subjects in the presence of two different ground surfaces, i.e., sloppy surface and plain surface.

Gait-mob-ACC-dataset [22]: It is the most challenging dataset that incorporates eight types of covariates along with speed variations. There are three sets of data such as Dataset #1, Dataset #2, and Dataset #3, which are captured from an accelerometer and kinetic sensor simultaneously. Here, inertial data from accelerometers are only included in the experiments. Among the three datasets, Dataset #1 contains 10 subjects, and each subject contain 100 samples. Out of 100 samples, half of the samples are collected from the fast walk and another half are collected from the normal walk. Dataset #2 has 50 subjects, with ten data samples for each subject. Dataset #3 has 50 subjects and 48 data samples from each subject. In particular, each subject requests to walk in eight different conditions, i.e., freestyle walking, hand in a pocket (left or right or both hands), holding a book either right or left hand, carrying loads either right or left hand.

### 4.2. Network Architecture

The proposed network has been built in an end-to-end fashion such that a gait sample is accepted from one end; then, it passes through sub-networks, which are tied together, and produces its identity at the other end. Each sub-network is connected with CNN, having four optimum numbers of convolutional layers in the order of a kernel regularization layer $\left(L_{2}\right)$, a ReLU activated layer followed by a max-pooling layer of size 2, and a dropout layer. Each layer has a filter attached, and the maximum depth is set as 32, 32, 64, and 128 in the order of four layers. An Adam optimizer is compiled with a learning rate of 0.001. The dropout layer is recognized to be the best option to reduce overfitting. Here, dropout is set at a rate of 0.5 after convolutional layers and 0.8 after the fusion layer to force other weights to neutralize. This leads to higher accuracy and a better understanding of the data. The weights of the convolutional layers and fully connected layers are initialized using the Kaiming initializer. The weighting factors α and β are manually tuned and set to 0.99 and 0.87, and a batch size of 32 is used for all experiments. The number of epochs for training is 200. The early stopping condition is set if no improvement is taking place after 50 consecutive epochs. The detailed parameters of the proposed single scale CNN network are given in Table 2. For multi-scale analysis, each input signal has a fixed dimension of 200 samples of length.

The experiments and the results are discussed on the following points:

1. Experiments on the effect of using the proposed weight update sub-network (Ws) into various CNN architectures.
2. Performance of the proposed methods in handling gait data collected under different covariate conditions.
3. Evaluation of the proposed method for identification and authentication.

### 4.3. Experiments on the Effect of Using the Proposed Weight Updated Sub-Networks (Ws) into Various CNN Architectures

The proposed Ws layer is integrated into various CNN backbones such as AlexNet [56], VGG14 [57], VGG16 [57], and ResNet50 [58], and we compare their performance for handling sensor-based gait signals in multi-scale analysis. To do so, all the fully connected layers are removed from each of the CNN backbones and replaced with Ws layers followed by a fully connected softmax layer. For example, in AlexNet, the layers after conv5 have been replaced with Ws. In both the architecture of VGG14 and VGG16, its single and triple FCN layers are replaced with Ws, respectively. In ResNet, the proposed layer is connected after the max-pooling layer to perform the task. From Table 3, it is observed that by employing Ws, the identification rate improves to 1–3% in each model. This is because each sub-network guides the extraction of more correlated features by focusing on the semantically relevant class-specific samples and ignoring the uncorrelated patterns. The performance of the proposed model is the best among all other models irrespective of different covariate conditions. Regarding the architectures, we find that ResNet50 performs comparably to the proposed model. Meanwhile, both VGG-14 and VGG-16 have similar performance in the identification rate, but VGG-16 shows a quite significant improvement in identification rate of 0.5% to 1.5% on the Gait-mob-ACC dataset. Furthermore, we observe that the performance of each model slowly declines as the size of sub-network *s* varies from 4 to 5. The best performance is recorded at the ensemble of 4 sub-networks.

### 4.4. Performance Evaluation of the Proposed Network under Different Covariate Conditions

The paper analyzes the performance of the proposed model on the most challenging dataset, i.e., Gait-mob-ACC [22], which contains possible co-variate factors ties in our daily life.

**Figure 4:**
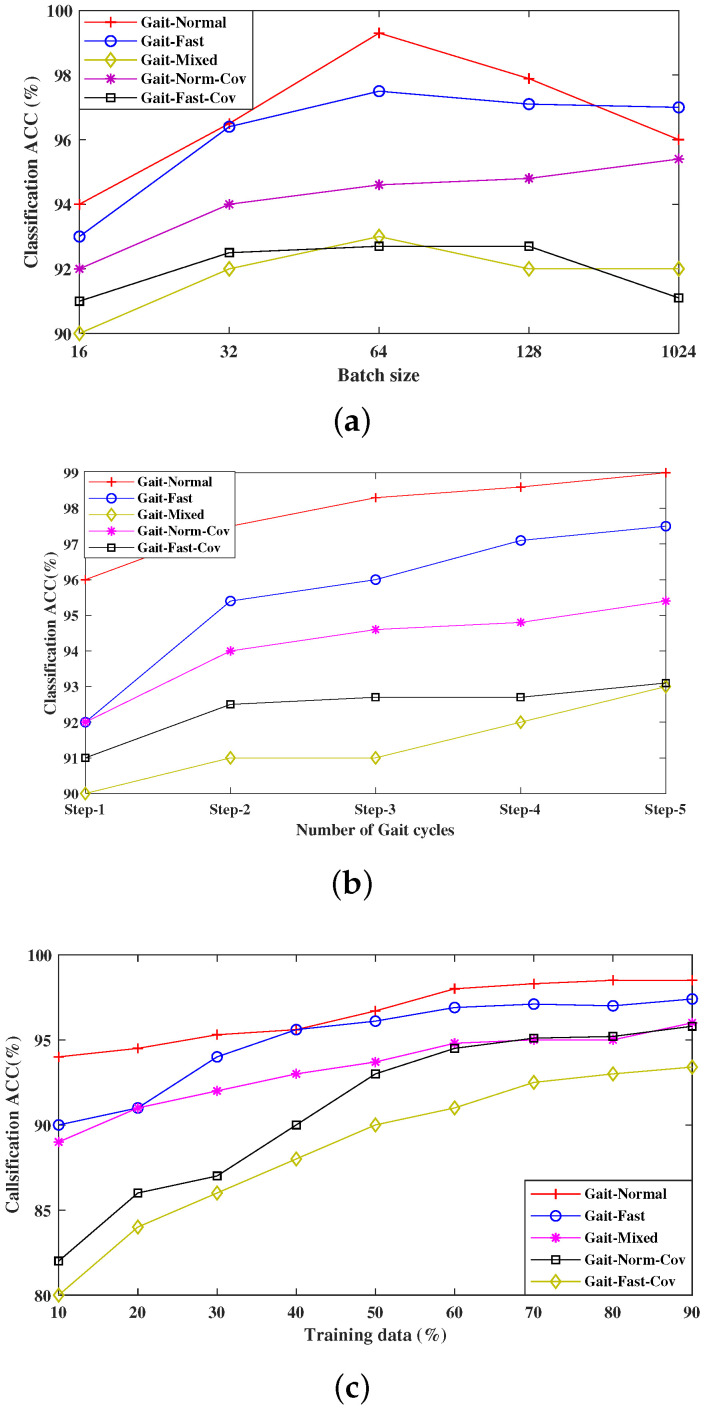
Performance evaluation of the proposed network in terms of accuracy on the five different types of gait sequences, with the influence of varying (**a**) batch size B (**b**) number of steps $N_{s}$ per gait cycle, (**c**) amount of training data.

To evaluate the proposed model on the above covariate conditions, the Gait-mob-ACC dataset is divided into five sub-datasets and named as Gait-normal, Gait-fast, Gait-mixed, Gait-fast-Covar, and Gait-normal-Covar, each having an equal number of subjects. Some experiments have been conducted with varying batch sizes, steps, and training samples to obtain the highest performance of the proposed model. The comparative results are shown in Figure 4a–c. It is observed from Figure 4a that the model obtains the best performance on different covariates by varying the batch size B. Increasing B from 16 to 32, the accuracy gradually improves from 94% to 94.6% in the normal walk, it improves around 0.45% more in both fast and mixed walks, and it improves 0.36% more in covariate conditions. However, when B reached more than 64, the accuracy is degraded to more than 0.07%. This is because after a certain increase of batch size, the overlapping may take the place of two gait cycles over two different persons, which shows erroneous results.

Another important setting for improving the performance is considering the number of walking cycles of a given model. The accuracy will increase with increasing the number of steps. It is shown from Figure 4 that at normal walking speed, the accuracy increased at a rate of 0.01–0.05%, whereas for fast walking, the rate of accuracy increase is about 0.1–0.3%. So, a higher step always gives better performance; however, higher steps for a person also entail a longer acquisition time, which we would rather avoid. Therefore, we restrict the number of steps $N_{s}$ = 2 in all the experiments, as it provides a good trade-off between accuracy and complexity across evaluations.

From Figure 4c, it is observed that the model obtains good recognition accuracy in all five cases, e.g., over 0.95 in a normal gait speed, 0.92 during fast walking, and 0.90 during mixed walking using 30% of training. Moreover, the model shows almost equal accuracy under normal and fast pace.

### 4.5. Identification and Authentication of Gait Based Bio-Metric System

The whole dataset is divided into two sub-datasets: a training and a test set. Both the training and the testing sets are made disjoint from each other. The experiment shows its performance in terms of identification and verification process. In the identification process, a identification rate (IR) has been used for rank-based classification. For the verification process, receiver operating characteristics (ROC) curves are obtained by plotting pairs of verification rates and false acceptance rates at various threshold values.

#### 4.5.1. Experimental Results on Identification

All the datasets for gait identification are processed through a common experimental set up. Here, each dataset is split into a training set considered as a gallery set, and the remaining is used for testing as the probe set. The distance scores between the whole gallery set are compared to each other to obtain the smallest score as IR. Table 4 demonstrates the Rank1 IR as compared to the other state-of-the-art methods on different benchmark datasets.

For the whuGait dataset, Dataset #2 achieves better performance as compared to Dataset #1 with an IR of more than 96%. This is because there are more samples per subject in Dataset #2 than in Dataset #1. It is also observed that both standalone networks CNN and LSTM perform approximately 0.3% better than parallely connected CNN and LSTM. One possible reason is that the parallel network may face over-fitting problems. Furthermore, it is noticeable that the performance of the CNN network is better than that of the LSTM network. $CNN_{fix}+LSTM$ and $LSTM_{fix}+CNN$ are both complementary networks of each other. Both are designed with parallel connection by fixing the parameter of one network and updating the other network. These two networks achieve an IR of approximately 93% and 92% on Dataset #1 and Dataset #2, respectively. The proposed network outperforms the other two networks such as IdNet and DeepconvLSTM with IR values of more than 2.34% and 2.05%, respectively. This is because the WMsCNN-Local model is a single-scale CNN architecture attached with a CWs sub-network that gives a competitive performance for its discriminative local feature analysis, whereas the multi-scale approach is incorporated with the proposed network modeled as (WMsCNN-Local-Global), which gives the best performance of around 99.96%.

In the IDNet dataset, all the collected gait samples are free style walking. Therefore, the IR values of all networks are quite high. The proposed approach achieves 99.96% IR.

In the OU-ISIR dataset, the LSTM network achieves better performance than the CNN+LSTM network in the presence of variation of gait sequences. For both Dataset #1 and #2, the proposed network obtains more than 73% IR. The result signifies that the proposed network can effectively handle variations of gait sequences better than other approaches.

In the Gait-mob-ACC database, six different covariates are incorporated in Dataset #3. It is the most challenging dataset, having speed variations from normal walk to fast walk. The last column of the table gives a detailed comparison. Deepconv competes with the proposed approach with a performance of less than 2%, but it achieves better results than other approaches. Our multi-scale approach can effectively handle complex features generated from covarite conditions such as both hands in the pocket, carrying loads, etc.

#### 4.5.2. Experiments on Authentication

The authentication task is performed by transforming the multi-class identification problem into a binary classification problem, which is based on the hypothesis of either positive acceptance or false acceptance. The authentication performance is evaluated by the metric of the average receiver operating characteristic (ROC) curve. It is created by plotting the true acceptance rate (TAR) against the false acceptance rate (FAR) at varying threshold settings. In the ROC curve, the value of FAR is set as 0.001% as the standard FAR for bio-metric authentication. The TAR and FAR are defined as

(15) $$TAR=\frac{True\;\;Positive}{True\;\;Positive+False\;\;Positive}$$

(16) $$FAR=\frac{False\;\;Positive}{False\;\;Positive+True\;\;Negative}$$

**Figure 5:**
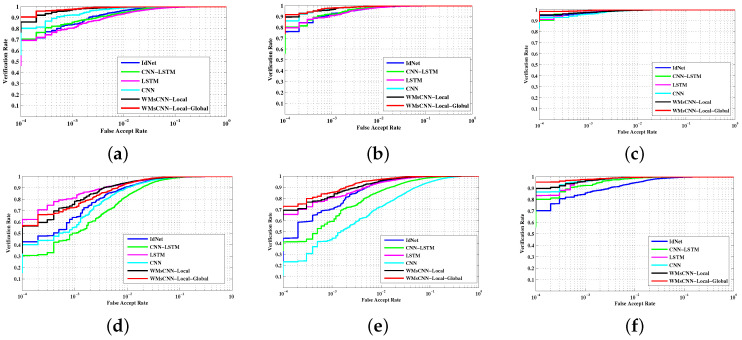
A comparative ROC curves of state-of-the-art deep learning networks: IDnet, CNN, LSTM, CNN+LSTM, and the proposed models. The performance of four benchmarks, each having different sub-datasets as referred in Table 1, is shown in (**a**–**f**). (**a**,**b**) refer to whuGait Dataset #1 and Dataset #2, respectively, (**c**) refers to the IdNet dataset, (**d**,**e**) refer to sub-dataset #1 and sub-dataset #2 of the OU-ISIR dataset, respectively, and (**f**) refers to the Gait-mob-ACC dataset.

To evaluate the system performance, the model is incorporated into different types of state-of-the-art methods. The experiments are conducted to examine the relative behavior of the false accept rate and the verification rate under different covariate conditions using (15) and (16). The ROC curves for the proposed method and the other state-of-the-art methods are plotted in Figure 5. The model achieves a higher verification rate at very low FARs. As we find from Figure 5b, the proposed network achieves limited improvement on the OU-ISIR dataset, while it has a notable performance on the whuGait dataset, IdNet datasets, and Gait-mob-ACC datasets as in Figure 5a,b,f respectively. In Figure 5d, the proposed network produces a very competing performance with LSTM, but later, it achieves equal performance with it when FAR is around 0.001. In response to the real environment, the Gait-mob-ACC dataset is considered, having multiple covariates along with speed variations from normal to fast. The performance of the proposed network is superior to others. After that, CNN finds it better than the other three networks. It can be observed from most figures that the multi-scale network uniformly outperforms overall networks, which may simply indicate that the multi-scale features are more discriminative by describing the detailed gait subdynamics. According to the above analysis of experimental results, we conclude that the combination of discriminate local features and global features is more suitable for the gait analysis on covariate conditions.

## 5. Discussion

A major contribution of this work is the joint use of the discriminative local features and global features to handle covariate factors and overall gait sequence variations, respectively. From Table 4, it is observed that WMsCNN-Local achieves good recognition accuracy using the benefits of Ws. However, combining all the local to global features further improves the recognition accuracy both in the identification and authentication module. It is reasonable that the global features only focus on overall gait cycle variations and ignore the multiple pose variations due to the effect of several covariates. Similarly, only local features ignore the overall variations. From the experimental results of Figure 5a–c, it is observed that the performance of the CNN network is quite appreciable because the features of CNN have more discriminable properties than the LSTM. Therefore, the proposed model (WMsCNN-Local-Global) outperforms as it captures CNN data at different time scales and combines them for a better representation of the feature sets. In addition, it is observed that direct features of LSTM are not appropriate for discriminating complex features such as gait, resulting in lowering the accuracy. Moreover, Table 4 shows the performance of the single-scale proposed model (WMsCNN-Local) and the multi-scale proposed model (WMsCNN-Local-Global), which gives the inference that the ensemble of sub-networks improves the performance of a single network. However, Table 4 reveals that more than 4 sub-networks degrade the performance of the overall network. Furthermore, in the evaluation of results, it is noticeable that some inconsistencies are found between the performances of the identification and authentication model. The performance of authentication is a little bit lower than the performance of identification. One possible reason for this is over-fitting, since only one test is used in the authentication process.

## 6. Conclusions

In this paper, an improved deep learning network is designed for gait recognition using smartphones. The novelty of the proposed approach lies in the feature extraction technique, which is based on a multi-scale signal approach and it is incorporated with a weight update feature sub-network to exploit significant local features. These sub-networks of each CNN architecture assign more weights to become discriminative feature regions for better classification. The significant of the local features from each scale are combined using a fusion network to achieve global-based features. The experiment performs on four benchmark datasets with different covariate conditions. The acquired results of the proposed framework reach an accuracy of 99.96% and 73.56% in the normal gait and most challenging gait database, respectively. The overall performance of the proposed model is superior compared to other state-of-the-art networks.

## Figures and Tables

**Table 1:** Details of four challenging datasets.

Database Name	No.	Number of Subjects	Sampling Rate	Challenges
**OU-ISIR**	#1 #2	745 408	100 Hz	A large database with fewer samples on each subject and each subject walks on a plain and sloppy surface
**whuGAIT**	#1 #2	118 20	50 Hz	
**Gait-mob-ACC**	#1 #2 #3	10 50 50		Variation of walking speed: normal and fast with seven different covariates: either hand/both hand in pocket, either hand holding book, and either hand with loadings
**IDNet**	-	50	100 Hz	Wear different shoe types and different clothes

**Table 2:** Detailed parameters of the proposed single-scale CNN network.

Layer Name	Input	Kernel Size	Number of Kernels	Feature Map	Number of Parameters
Conv1_1	200×1×6	9×1	32	192×1×32	1760
MaxPool1	192×1×32	2×1	/	96×1×32	
Conv2_1	96×1×32	5×1	64	92×1×64	10,304
Conv2_2	92×1×64	5×1	128	46×1×128	41,088
MaxPool2	46×1×128	2×1	/	23×1×128	
Conv3_1	23×1×128	3×1	128	21×1×128	49,280

**Table 3:** Rank-1 and Rank-5 identification rates, and verification rate (VR) of different gait datasets are reported by integrating 2/3/4/5 numbers of the Ws sub-network layers into various CNN architectures at the presence of different time scales (τ). Bold font indicates the best performance.

sub-Networks (s)		whuGait				IDnet				OU-ISIR				Gait-Mob-ACC		
		Rank1	Rank-5	VR		Rank-1	Rank-5	VR		Rank-1	Rank-5	VR		Rank-1	Rank-5	VR
		Id	Id	(FAR = $10^{-3}$)		Id	Id	(FAR = $10^{-3}$)		Id	Id	(FAR = $10^{-3}$)		Id	Id	(FAR = $10^{-3}$)
2 (τ=4,5)	AlexNet	78.69	82.94	0.79		81.98	83.99	0.79		55.89	61.82	0.43		75.88	80.75	0.74
	VGG-14	83.66	86.43	0.83		86.24	90.91	0.86		57.76	61.83	0.44		75.94	79.04	0.74
	VGG-16	84.56	87.06	0.83		86.31	91.65	0.87		57.88	62.95	0.44		76.32	79.99	0.75
	ResNet-50	**93.06**	**95.83**	**0.88**		**92.95**	**94.09**	**0.91**		**67.47**	**70.54**	**0.49**		**86.21**	**89.77**	**0.91**
	CWs-AlexNet	80.98	85.91	0.82		84.24	93.46	0.81		56.91	61.98	0.43		78.88	83.18	0.77
	CWs-VGG14	85.87	90.05	0.85		88.06	93.32	0.88		60.78	64.21	0.46		83.19	88.06	0.79
	CWs-VGG16	86.33	91.87	0.86		89.87	94.54	0.89		61.43	65.32	0.47		84.67	88.42	0.8
	CWs-ResNet50	95.04	97.76	0.91		96.11	97.97	0.93		69.53	73.89	0.51		89.55	93.67	0.94
	proposed	**95.32**	**98.08**	**0.92**		**96.34**	**98.56**	**0.93**		**70.34**	**75.85**	**0.52**		**91.32**	**94.43**	**0.94**
3 (τ=3,4,5)	AlexNet	89.01	92.64	0.81		88.87	92.57	0.84		59.56	61.84	0.45		80.01	83.65	0.87
	VGG14	91.32	94.54	0.82		92.01	95.36	0.87		61.19	65.92	0.47		83.88	87.73	0.88
	VGG16	91.78	95.81	0.83		92.42	95.75	0.88		61.76	64.20	0.46		84.88	88.78	0.89
	ResNet50	**93.54**	**97.47**	**0.88**		**96.24**	**97.96**	**0.94**		**68.54**	**72.86**	**0.51**		**89.65**	**93.64**	**0.92**
	CWs-AlexNet	90.54	93.04	0.83		90.76	94.35	0.85		61.82	65.47	0.47		83.71	85.45	0.90
	CWs-VGG14	93.12	96.13	0.89		94.02	97.61	0.89		64.89	68.78	0.48		87.21	91.65	0.91
	CWs-VGG16	93.03	96.43	0.89		94.24	97.86	0.90		65.56	69.35	0.49		88.45	93.67	0.93
	CWs-ResNet50	96.32	98.53	0.93		98.24	100	0.96		72.86	76.59	0.53		94.01	98.15	0.96
	Proposed	**97.36**	**99.78**	**0.94**		**99.96**	**100**	**0.96**		**73.38**	**77.51**	**0.54**		**94.05**	**98.64**	**0.96**
4 (τ=2,3,4,5)	AlexNet	87.43	92.56	0.83		86.21	90.64	0.83		53.54	58.43	0.40		72.88	77.67	0.86
	VGG14	87.43	91.01	0.84		88.12	92.89	0.86		60.19	64.84	0.46		80.32	84.43	0.88
	VGG16	88.34	92.43	0.84		88.51	93.30	0.86		60.48	65.48	0.46		82.98	85.13	0.89
	ResNet50	**91.89**	**95.94**	**0.88**		**90.89**	**94.14**	**0.89**		**64.97**	**69.99**	**0.50**		**86.16**	**90.98**	**0.92**
	CWs-AlexNet	90.34	95.89	0.86		88.13	93.03	0.85		55.76	60.20	0.41		76.71	80.51	0.87
	CWs-VGG14	91.03	95.96	0.86		90.07	95.46	0.87		64.98	68.78	0.48		84.23	88.89	0.92
	CWs-VGG16	91.89	96.65	0.87		90.45	94.55	0.88		65.16	69.98	0.49		86.89	91.78	0.94
	CWs-ResNet50	94.12	98.27	0.9		94.39	97.06	0.92		69.97	73.32	0.52		91.13	91.98	0.95
	Proposed	**97.01**	**99.75**	**0.94**		**99.93**	**100**	**0.96**		**73.56**	**78.84**	**0.55**		**94.88**	**99.14**	**0.97**
5 (τ=1,2,3,4,5)	AlexNet	79.45	83.65	0.76		79.63	84.32	0.82		52.17	56.99	0.41		77.44	80.21	0.81
	VGG14	81.69	84.32	0.78		86.33	90.56	0.82		53.11	57.42	0.4		79.64	82.43	0.83
	VGG16	81.94	84.87	0.79		87.44	91.21	0.83		54.98	57.96	0.4		81.01	85.78	0.85
	ResNet50	**90.15**	**94.47**	**0.89**		**92.31**	**97.09**	**0.9**		**62.33**	**69.08**	**0.50**		**88.56**	**92.11**	**0.90**
	CWs-AlexNet	83.17	85.54	0.79		82.54	87.54	0.83		54.12	58.73	0.42		80.32	84.04	0.82
	CWs-VGG14	89.12	91.35	0.85		89.32	93.76	0.87		61.41	64.56	0.45		84.36	87.55	0.89
	CWs-VGG16	89.54	92.98	0.85		91.07	93.86	0.88		61.59	65.32	0.46		86.14	90.76	0.91
	CWs-ResNet50	93.32	97.89	0.91		95.89	97.32	0.91		65.76	71.34	0.50		90.12	94.32	0.93
	Proposed	**96.38**	**98.64**	**0.93**		**98.76**	**99.32**	**0.94**		**72.16**	**76.18**	**0.53**		**93.89**	**98.56**	**0.96**

**Table 4:** Comparison of state-of-the-art methods on different benchmark datasets in terms of Rank-1 identification rate.

Methods	whuGait Dataset1 (118 Subjects)	whuGait Dataset2 (20 Subjects)	IDNet Dataset (50 Subjects)	OU-ISIR Dataset (745 Subjects)	OU-ISIR Dataset2 (408 Subjects)	Gait-Mob-ACC Dataset3 (50 Subjects)
IdNet [23]	92.91%	96.78%	99.58%	44.29%	46.20%	74.75%
CNN [34]	92.89%	97.02%	99.71%	40.60%	47.14%	90.2%
LSTM [30]	91.88%	96.98%	99.46%	66.36%	65.32%	81.65%
DeepConv [35]	92.25%	96.80%	99.24%	37.33%	41.32%	86.23%
CNN+LSTM [24]	92.51%	96.82%	99.61%	34.28%	53.96%	89.22%
$CNN_{fix}$+LSTM [24]	92.94%	97.04%	99.64%	-	-	-
CNN+$LSTM_{fix}$ [24]	93.52%	97.33%	99.75%	-	-	-
WMsCNN-Local	93.36%	98.28%	99.81%	65.74%	72.13%	90.49%
WMsCNN-Local-Global	95.75%	98.98%	99.96%	73.56%	76.42%	94.71%

## Data Availability

Not applicable.
